# Assessment of colonic interposition graft using indocyanine green: expanding fluorescence imaging techniques in gastrointestinal surgery

**DOI:** 10.1093/jscr/rjaf816

**Published:** 2025-10-14

**Authors:** Isabella Zappala, Mina Sarofim, Arthur Richardson, Toufic El-Khoury

**Affiliations:** Department of Surgery, Westmead Hospital, Cnr Hawkesbury and Darcy Roads, Westmead, Sydney, NSW 2145, Australia; Faculty of Medicine and Health, The University of Sydney, Camperdown, Sydney, NSW 2050, Australia; Department of Surgery, Westmead Hospital, Cnr Hawkesbury and Darcy Roads, Westmead, Sydney, NSW 2145, Australia; Faculty of Medicine and Health, The University of Sydney, Camperdown, Sydney, NSW 2050, Australia; Department of Surgery, Westmead Hospital, Cnr Hawkesbury and Darcy Roads, Westmead, Sydney, NSW 2145, Australia; Faculty of Medicine and Health, The University of Sydney, Camperdown, Sydney, NSW 2050, Australia; Department of Surgery, Westmead Hospital, Cnr Hawkesbury and Darcy Roads, Westmead, Sydney, NSW 2145, Australia; Faculty of Medicine and Health, The University of Sydney, Camperdown, Sydney, NSW 2050, Australia

**Keywords:** indocyanine green, colonic interposition graft, perfusion assessment, anastomosis, anastomotic leak, oesophagectomy with colonic conduit

## Abstract

Indocyanine green (ICG) fluorescence is a well-established intra-operative imaging technique used to assess adequate tissue perfusion in bowel anastomoses. Current evidence shows it to be a cost-effective and safe procedure effective in reducing anastomotic leak rates in colorectal anastomoses. However, the application of this imaging method to assess viability of colonic conduits in the upper gastrointestinal tract is rarely accounted for in the literature. Colonic interposition grafting following oesophagectomy is a complex procedure that carries many risks including conduit ischaemia or anastomotic leak, which are life-threatening complications. We present a case of a 55-year-old gentleman who underwent delayed oesophageal reconstruction with a colonic interposition graft following oesophageal conduit necrosis complicating an Ivor-Lewis oesophagectomy. To decrease risk of morbidity, ICG was administered intravenously to assess sufficient perfusion at the oesophago-colonic and entero-colonic anastomotic sites.

## Introduction

The surgical approach to major oncological resections of the gastrointestinal (GI) tract has dramatically improved over time to minimize significant morbidity and mortality [1]. However, these procedures remain high-risk due to complications of anastomotic leak (AL). For some patients, AL can be a fatal complication, lengthen hospital stay, and negatively affect morbidity and mortality beyond 30 days of initial operation [2]. Rates of AL depend upon patient and operative factors including site of anastomosis and range from ~1%–18% in colorectal surgery and 3%–25% in oesophageal anastomoses [3, 4]. Monitoring C-reactive protein (CRP) levels post-operatively may be an early predictor of AL and guide the need for definitive diagnosis and management [3].

Intra-operatively, rates of AL have reduced following assessment of adequate perfusion at the anastomotic site [5]. Several imaging modalities have evolved to provide visual or objective markers of perfusion before creation of an anastomosis. The most common method is ICG with fluorescence angiography due to its unique absorption and emission spectrum, non-toxic profile and established wide application in GI surgery [6]. ICG is a water-soluble fluorescent dye that binds proteins in the lymphovasculature within 45s of intravenous injection [7]. Once exposed to fluorescence imaging, the dye provides a visual marker of adequate blood and lymph flow and is useful intra-operatively as it does not interfere with human tissue and bodily fluid auto-fluorescence [7]. A systematic review in 2023 showed significant reduction in AL rates in colonic anastomoses following intra-operative visualisation of ICG [8]. Similarly, in gastro-oesophageal junction surgery, ICG fluorescence has shown to significantly reduce the incidence of AL [9].

However, there is very limited application of ICG in colonic interposition grafts used in oesophageal reconstruction surgery where there is absence of a viable gastric conduit. These salvage procedures have high rates of multiple ALs, up to 37.5% that contribute to its high morbidity and mortality [10]. Intra-operative perfusion assessment with ICG may curtail the high risk of AL in these procedures. We present a unique and successful case of perfusion assessment using ICG fluorescence angiography of a colonic interposition graft in oesophageal reconstruction after AL of the gastro-oesophageal anastomosis formed in an Ivor-Lewis procedure.

## Case report

A 55-year-old gentleman with a background history of gastro-oesopheageal adenocarcinoma, BMI of 39.3 kg/m^2^, and a previous 25-pack-year smoking history presented to hospital for elective retrosternal reconstruction of the distal oesophagus using a colonic interposition graft. This was a secondary procedure performed five months after an Ivor-Lewis oesophagectomy and insertion of feeding jejunostomy, which was complicated by anastomotic leak and necrosis of the gastric conduit. The reconstruction was performed with intra-operative indocyanine green fluorescence (ICG) angiography to ensure adequate perfusion of the colon prior to anastomoses.

The procedure was performed by opening three compartments, a left neck incision to access the proximal oesophagus, midline sternotomy to allow anastomosis and a midline laparotomy for conduit preparation. In the abdomen, the right and transverse colon was fully mobilized and divided at the terminal ileum with a linear stapler after Bulldog clamp occlusion of the ileocolic pedicle confirmed no compromise. The ileocolic pedicle was divided with suture ligation to allow the entire ileum and caecum to adequately reach the neck. The middle colic pedicle was carefully preserved.

In the neck, a left sternocleidomastoid incision allowed dissection to the oesophagus. The left recurrent laryngeal nerve and brachiocephalic trunk were identified and protected. The oesophagus was dissected from the trachea-oesophageal groove. The distal oesophagus was not dissected free due to adherence of scar tissue to the prevertebral fascia. Division of the proximal oesophagus was made posterior to the aortic arch and was the planned proximal end of for the new interposition graft.

**Figure 1:**
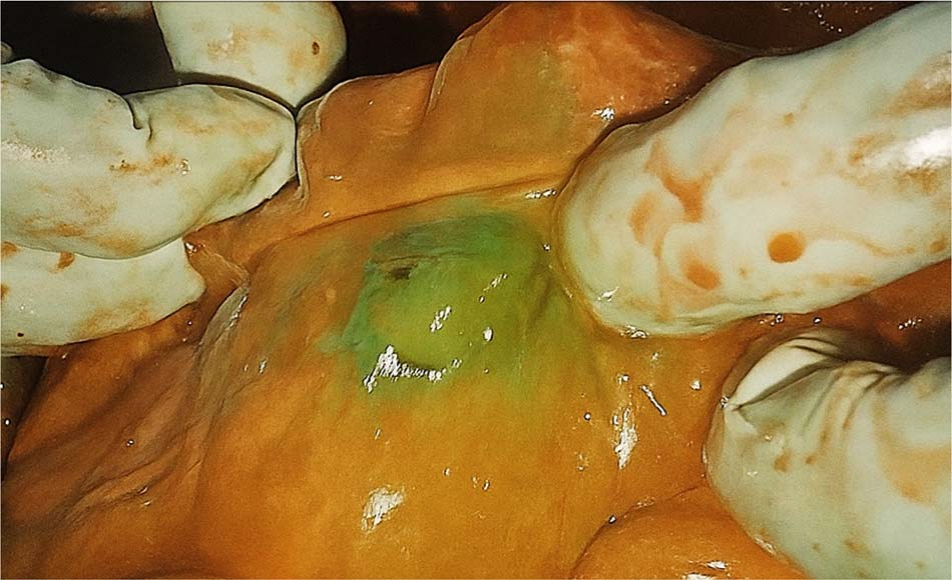
Intra-operative ICG fluorescence of the caecum (distal most aspect of colonic conduit) indicating satisfactory perfusion.

The colon was carefully delivered to the retrosternal space. Blood flow at the caecum was assessed using ICG angiography (Fig. 1). Within 40s, uniform fluorescence was visualised at the caecum (which would anastomose to the oesophagus), at similar intensity as the remainder of the large bowel. A colotomy at the base of the caecum was made to insert the anvil of a 21 mm circular stapler. An end-to-side oesophago-colonic anastomosis was performed. Partial caecectomy with a linear stapler was then performed to resect the appendix, distal terminal ileum and the colotomy created for entry of the circular stapler. The anastomosis was reinforced with a second layer of interrupted 3/0 PDS. A leak test was performed with nasogastric insufflation, which was negative. The colonic conduit was divided at the transverse colon with a linear stapler and a side-to-side antiperistaltic ileocolic anastomosis was fashioned with two further firings of the linear stapler. A side-to-side isoperistaltic colo-jejunal anastomosis was then fashioned with linear stapler and interrupted 3/0 PDS to close the common enterotomy. His existing feeding jejunostomy was left in situ.

There were no immediate complications post-operatively. The patient had one fever on post-operative day 2. Blood cultures returned *Proteus hauseri* and he was treated with IV meropenem for 7 days. Day 3 CRP was recorded as 107 mg/L, however, this continued a daily downward trend to 6 mg/L. The patient was upgraded to a regular diet from jejunostomy feeds on post-operative day 10. He was discharged home on post-operative day 20 with no clinical or radiological concerns of anastomotic leak. He will follow up for removal of the feeding jejunostomy.

## Discussion

This case demonstrates the role of ICG fluorescence in mitigating intra-operative risk of AL by providing an adequate assessment of conduit perfusion prior to oesophageal reconstruction. To date, there are only five case reports worldwide that describe successful application of ICG in this procedure [11]. In all five cases, there were no adverse effects of ICG administration. Intra-operative visualisation of adequate fluorescent flow was clinician dependent in all cases and areas of uniform perfusion were selected for creation of the anastomosis. Each study recorded no AL on post-operative review and highlight the efficacy of qualitative perfusion markers intra-operatively [11].

There are patient-related risk factors that independently correlate to the development of AL regardless of intra-operative optimisation. These include male sex, increased body mass index (BMI) >30 kg/m^2^ and location of the lesion in the GI tract [5]. A heavy smoking history of >40 pack-years has a strong association with AL due to development of tobacco-associated microvascular disease that impedes tissue healing [12]. The patient in this study had accumulated multiple risk factors for AL including factors that were independent of any intra-operative risk, including male sex, high BMI, and a 40-pack-year smoking history. Studies also found neoadjuvant chemotherapy to be a major and independent pre-operative risk factor for increased AL rates [5]. Our patient completed four cycles of neoadjuvant chemotherapy for gastro-oesophageal junction adenocarcinoma prior to initial procedure. Although intra-operative ICG assessment has shown to reduce the risk of AL, additional patient and pre-operative factors may counterbalance the overall risk of AL.

Furthermore, the route of oesophageal reconstruction influences the risk of AL [13]. Reconstruction is performed by posterior mediastinal, retrosternal or subcutaneous approach, with each possessing their own risk profile. A meta-analysis in 2023 investigated post-operative complications of patients following oesophagectomy by posterior mediastinal and restrosternal approach [14]. There were significantly lower rates of AL in the posterior mediastinal vs retrosternal approach, however, retrosternal approaches had lower incidences of pneumonia. Additional pulmonary complications and overall mortality of oesophageal reconstruction was unchanged between the two approaches [14]. Decision to proceed by retrosternal route in our patient was based on attempts to reduce risk of pneumonia due to a previous history of hypoxic and hypercapnoeic respiratory failure following initial Ivor-Lewis oesophagectomy as well as surgeon preference. Perfusion assessment was performed before establishing the colo-oesophageal anastomosis, reducing the risk of AL reported in the retrosternal approach.
